# Efficacy and Tolerability of Sodium Zirconium Cyclosilicate or Patiromer in Managing Hyperkalemia: A Systematic Review and Meta-Analysis

**DOI:** 10.34067/KID.0000001173

**Published:** 2026-03-11

**Authors:** Wannasit Wathanavasin, Solos Jaturapisanukul, Charat Thongprayoon, Wisit Cheungpasitporn

**Affiliations:** 1 Department of Medicine, Nephrology Unit, Charoenkrung Pracharak Hospital, Bangkok Metropolitan Administration, Bangkok, Thailand; 2 Division of Nephrology and Hypertension, Department of Medicine, Mayo Clinic, Rochester, Minnesota; 3 Nephrology and Renal Replacement Therapy Division, Department of Internal Medicine, Faculty of Medicine Vajira Hospital, Navamindradhiraj University, Bangkok, Thailand

**Keywords:** CKD, hyperkalemia

## Abstract

**Key Points:**

- Both sodium zirconium cyclosilicate and patiromer effectively lower serum potassium and provide clinical benefits.
- Sodium zirconium cyclosilicate is associated with higher risk of edema. Patiromer is associated with the increased risk of constipation and hypokalemia.

**Background:**

Sodium zirconium cyclosilicate (SZC) and patiromer have recently been approved for the treatment of hyperkalemia. However, their efficacy in lowering serum potassium according to the phase of intervention, safety profile, and impact clinical outcomes remains to be fully defined. This study aims to assess their efficacy and tolerability in the management of hyperkalemia.

**Methods:**

A comprehensive search of PubMed, Scopus, and Cochrane Central Register of Controlled Trials was conducted up to June 6, 2025. Eligible studies included randomized controlled trials reporting the effects of SZC and patiromer on laboratory parameters, clinical outcomes, and safety profiles. Meta-analyses were synthesized using a random-effects model, with results presented as weighted mean differences (WMD) or relative risks (RR), along with 95% confidence intervals (CI).

**Results:**

Nineteen randomized controlled trials involving 3627 patients (1911 on SZC; 1716 on patiromer) were analyzed. During the initial 48–72 hours, only SZC significantly reduced serum potassium levels (WMD, −0.45 mEq/L; 95% CI, –0.61 to –0.29). At the end of treatment, both agents effectively lowered serum potassium (SZC: WMD, –0.50 mEq/L; 95% CI, –0.63 to –0.38; patiromer: WMD, –0.36 mEq/L; 95% CI, –0.51 to –0.21). SZC significantly normalized serum potassium and reduced hyperkalemia incidence, while patiromer enabled renin-angiotensin-aldosterone system inhibitor optimization by supporting target dosing and reducing discontinuation due to hyperkalemia. However, SZC was associated with a higher incidence of edema (RR, 2.92; 95% CI, 1.08 to 7.90) and patiromer with increased rates of constipation (RR, 2.96; 95% CI, 1.21 to 7.22) and hypokalemia (RR, 1.46; 95% CI, 1.05 to 2.03).

**Conclusions:**

Both SZC and patiromer effectively lower serum potassium and provide clinical benefits, with distinct safety considerations. Therapy selection should be guided by treatment goals and the patient's individual risk profile.

## Visual Abstract

**Figure:**
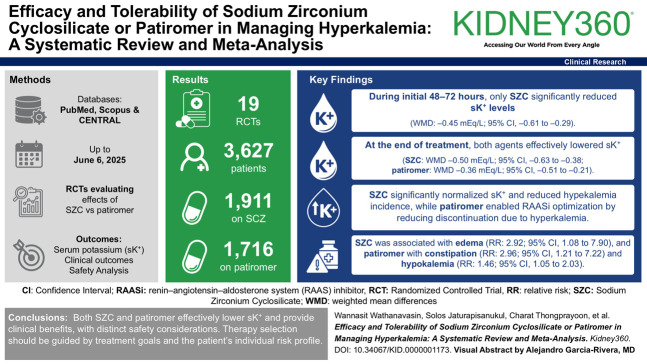

## Introduction

Hyperkalemia, defined as an elevated serum potassium concentration >5.0 or 5.5 mEq/L, can lead to potentially life-threatening consequences.^1^ Since the kidneys play a central role in regulating total body potassium, hyperkalemia often reflects impaired renal potassium handling.^2^ In addition to patients with CKD, individuals with diabetes mellitus (DM) or heart failure are also at increased risk for hyperkalemia, especially when treated with renin-angiotensin-aldosterone system (RAAS) inhibitors.^3^

In real-world clinical settings, several issues complicate hyperkalemia management, including: (*1*) dietary potassium restriction is commonly recommended, which may deprive patients of essential, nutrient-rich, heart-healthy foods^4,5^; (*2*) clinicians often reduce or discontinue RAAS inhibitors, preventing optimal therapeutic doses crucial for slowing CKD progression and improving cardiovascular outcomes^6^; and (*3*) in patients receiving hemodialysis, potassium accumulation after a long interdialytic interval increases the risk of arrhythmias and sudden cardiac death, especially when exposed to low-potassium dialysate during hemodialysis.^7,8^ Thus, balancing prevention of hyperkalemia with optimal management is crucial, albeit challenging.

**Figure 1:**
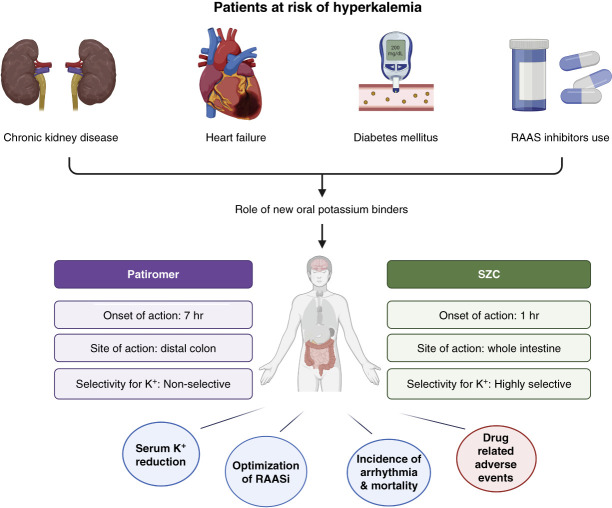
**A conceptual framework outlining the key characteristics of new oral potassium binders, patiromer, and sodium zirconium cyclosilicate, in managing patients with hyperkalemia, along with the study's objective to evaluate their efficacy and tolerability in clinical trials. **RAAS, renin-angiotensin-aldosterone system; SZC, sodium zirconium cyclosilicate.

The Food and Drug Administration and European Medicines Agency approval of two potassium-binding agents, sodium zirconium cyclosilicate (SZC) and patiromer, offers options to bridge the gap in hyperkalemia treatment. These oral agents are nonabsorbable polymers that bind potassium within the gastrointestinal (GI) tract, promoting fecal potassium excretion. Although both SZC and patiromer are effective for lowering serum potassium, recent studies warrant a comprehensive synthesis to clarify their role in RAAS inhibitor optimization and long-term clinical outcomes (Figure 1). Therefore, we conducted this study to assess the efficacy and tolerability of SZC or patiromer in managing hyperkalemia and to investigate efficacy by phase of intervention and across comorbidities.

## Methods

This systematic review was conducted in compliance with the 2020 Preferred Reporting Items for Systematic Reviews and Meta-Analyses guidelines for reporting interventions. The protocol was registered in the International Prospective Register of Systematic Reviews (PROSPERO; CRD420251125764).

### Search Strategy

A predefined search strategy was used by two independent reviewers (Wannasit Wathanavasin and Solos Jaturapisanukul) to identify relevant literature from inception to June 6, 2025, using PubMed, Scopus, and the Cochrane Central Register of Controlled Trials, without language or publication date restriction. The full search strategy is presented in Supplemental Table 1.

### Eligibility Criteria

We included randomized controlled trials that: (*1*) involved patients aged 18 years or older with hyperkalemia or at high risk for hyperkalemia; (*2*) compared patiromer or SZC with a control (placebo or standard of care) for managing hyperkalemia; and (*3*) reported at least one of the following outcomes: change in serum potassium during correction and maintenance phases, proportions of responders according to study-defined criteria, proportion of patients achieving normal serum potassium levels, proportion reaching the target RAAS inhibitors dose, change in serum bicarbonate during maintenance phase, incidence of cardiac arrhythmia, mortality, and safety outcomes. Exclusion criteria were: (*1*) reviews, editorials, conference abstracts, and responses to letters and (*2*) nonhuman studies.

### Data Extraction

Two reviewers (Wannasit Wathanavasin and Solos Jaturapisanukul) independently screened the titles/abstracts and assessed the full-text articles. Disagreements were resolved through discussion with a third author (Wisit Cheungpasitporn). For duplicate studies of the same patient population, the larger study was used as the main data source. Extracted variables included: first author, publication year, country, number of participants, patient characteristics, intervention type/dose/frequency, outcomes, follow-up duration, and risk of bias. For continuous outcomes, we collected either mean and mean change values with SDs, or median values with their interquartile ranges.

### Assessments of Quality and Risk of Bias

The risk of bias was independently assessed by two reviewers (Wannasit Wathanavasin and Solos Jaturapisanukul) using the Cochrane risk of bias tool for randomized trials (RoB 2), per Cochrane's 2022 recommendations. Discrepancies were resolved by the third author (Wisit Cheungpasitporn).

### Statistical Analyses

Statistical analyses were conducted using Stata version 18 (StataCorp, College Station, TX). A two-tailed *P* value <0.05 was considered statistically significant. Random-effects meta-analyses generated pooled effect estimates with 95% confidence intervals (CI). Dichotomous outcomes were summarized as pooled relative risks (RRs) with 95% CI. Continuous outcomes were analyzed using weighted mean differences (WMD) with 95% CI.^9^ Heterogeneity was assessed using the *I*^2^ statistic and *Q* test *P* value. Prespecified subgroup analyses were performed by continent, comorbidities, phase of intervention, and risk of bias. Meta-regressions evaluated whether study and patient characteristics were associated with heterogeneity. A sensitivity analysis was conducted using the leave-one-out approach. A forest plot was used to visually display heterogeneity across the included studies. Publication bias was assessed using funnel plots and the Egger’s test.

## Results

### Search Results

**Figure 2:**
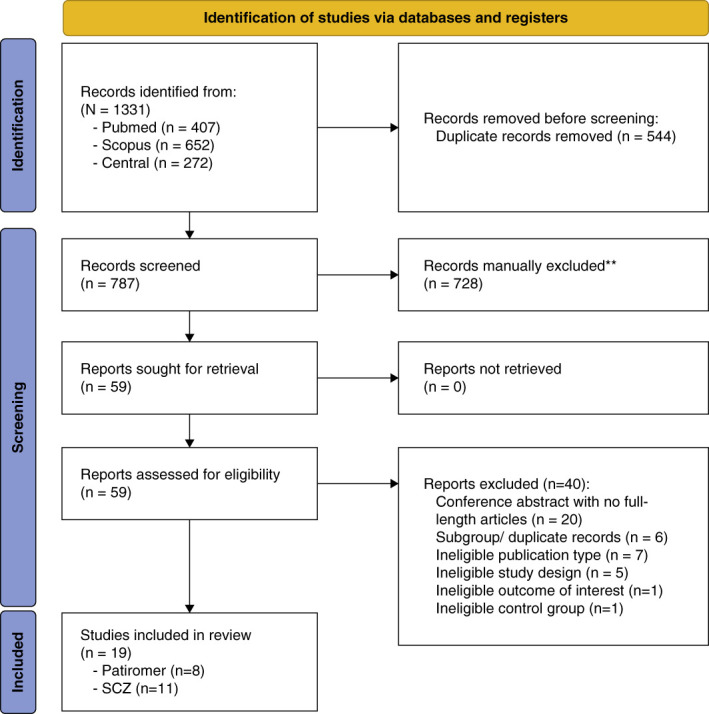
**PRISMA 2020 flow diagram**. PRISMA, Preferred Reporting Items for Systematic Reviews and Meta-Analyses

A search of PubMed, Scopus, and Cochrane Central Register of Controlled Trials databases identified 1331 potentially relevant articles. After removing 544 duplicates, the titles and abstracts of 787 articles were screened based on predefined eligibility criteria. Of the 59 articles, full texts were assessed. Forty-one articles were excluded, with reasons outlined in Figure 2. Finally, 19 studies were included in the analysis—eight on patiromer^10–17^ and 11 on SZC^18–28^ (Figure 2).

### Study Characteristics

Table 1 summarized the characteristics of the 19 included studies involving 3627 patients with hyperkalemia or at risk for the condition. Six focused exclusively on hemodialysis patients. The mean age was 63 years; 59% were male. Comorbidities included CKD in 76%, DM in 51%, and heart failure in 57% of patients. At baseline, 79% were receiving a RAAS inhibitor. The mean baseline serum potassium was 5.2 mEq/L, and the mean baseline eGFR, excluding hemodialysis patients, was 51 ml/min per 1.73 m^2^.

**Table 1:** Characteristics of the studies included in the systematic review

No	Author	Yr	Country	*N*	Age (yr)	Baseline Serum Potassium (mEq/L)	Baseline eGFR (ml/min per 1.73 m^2^)	CKD (%)	DM (%)	Heart Failure (%)	RAAS Inhibitors Use (%)	Diuretic Use (%)	Treatment (Drug Type, Dose, Frequency)	Comparator	Outcomes of interest^a^	Treatment Period	Risk of Bias
1	Pitt B. (PEARL-HF)	2011	Multicountry	105	68±10	4.7±0.4	81.2±33.6	55	32	100	90	0.7	Patiromer	Placebo	^b^ ^,^ ^c^ ^,^ ^d^ ^,^ ^e^ ^,^ ^f^	4 wk	High
													- I Phase: 15 g twice a d (30 g/d)				
													- M phase: 15 g twice a d (30 g/d)				
2	Kosiborod M. (HARMONIZE)	2014	Multicountry	258	64±13	5.6±0.4	46.3±30.5	66	66	36	70	NA	SZC	Placebo	^b^ ^,^ ^c^ ^,^ ^e^ ^,^ ^f^ ^,^ ^g^	4 wk	Some concerns
													- I Phase: 10 g thrice a d×48 h				
													- M phase: 5, 10 g, 15 g/d				
3	Ash SR	2015	USA	90	71±9	5.1±0.4	55.8±23.7	100	56	NA	62	33	SZC	Placebo	^b^ ^,^ ^e^ ^,^ ^h^ ^,^ ^f^	1 wk	Low
													- 0.3, 3, or 10 g thrice a d×48 h up to 4 d (max 12 doses)				
4	Packham DK	2015	Multicountry	753	66±12	5.3	NA	75	60	40	67	NA	SZC	Placebo	^b^ ^,^ ^c^ ^,^ ^f^ ^,^ ^g^	12 d	Some concerns
													- I Phase: 1.25, 2.5, 5, or 10 g thrice a d×48 h				
													- M phase: 1.25, 2.5, 5, or 10 g thrice a d until 12 d				
5	Weir MR	2015	Multicountry	107	65±9	5.9±0.5	38.8±20.5	100	63	46	100	51	Patiromer	Placebo	^b^ ^,^ ^c^ ^,^ ^d^ ^,^ ^i^ ^,^ ^f^	4 wk	Some concerns
													4.2 g twice a d if serum potassium 5.1–<5.5 and				
													8.4 g twice a d if serum potassium 5.5–<6.5				
6	Agarwal R (AMBER)	2019	Multicountry	295	68±12	4.7±0.4	35.7±7.4	100	50	45	100	99	Patiromer - 8.4 g/d	Placebo	^c^ ^,^ ^d^ ^,^ ^e^ ^,^ ^f^	12 wk	Low
7	Fishbane S	2019	Multicountry	196	58±14	5.9±0.6	- (HD patients)	100	NA	NA	NA	NA	SZC - 5–15 g once daily on nonhemodialysis days	Placebo	^b^ ^,^ ^e^ ^,^ ^f^ ^,^ ^g^	8 wk	Low
8	Kashihara N	2020	Japan	103	73±8	5.6±0.4	26.1±14.9	76	60	14	78	36	SZC - 5 g thrice a d or 10 g thrice a d for 48 h	Placebo	^b^ ^,^ ^i^ ^,^ ^f^ ^,^ ^g^	1 wk	Low
9	Peacock WF (ENERGIZE)	2020	Multicountry	70	59±14	6.4±0.7	NA	36	30	NA	36	NA	SZC 10 g up to three doses within 10 h	Placebo	^b^ ^,^ ^c^ ^,^ ^e^ ^,^ ^f^ ^,^ ^g^	24 h	Some concerns
10	Butler J (DIAMOND)	2022	Multicountry	878	67±10	4.6±0.3	63.0±22.0	47	41	100	100	NA	Patiromer - Start at 8.4 g/d and up-titrate as necessary up to 25.2 g/d	Placebo	^b^ ^,^ ^c^ ^,^ ^d^ ^,^ ^e^ ^,^ ^f^ ^,^ ^i^	27 wk	Some concerns
11	Jaques DA	2022	Switzerland	48	66±19	5.2±0.8	- (HD patients)	100	43	NA	52	33	Patiromer - 16.8 g/d (non-HD days)	SPS 15 g/d (non-HD days)	^c^ ^,^ ^e^ ^,^ ^f^	4 wk	Low
12	Ni Z (DIALYZE China)	2023	China	134	55±11	5.9±0.6	- (HD patients)	100	NA	NA	NA	NA	SZC	Placebo	^b^ ^,^ ^e^ ^,^ ^f^ ^,^ ^g^	8 wk	Low
													- I Phase: 5 g/d (nonhemodialysis days)				
													- M phase: 5–15 g/d (nonhemodialysis days)				
13	Ash SR (NEUTRALIZE)	2024	USA	37	63±14	5.5±0.4	21.7±12	89	70	0	74	NA	SZC	Placebo	^b^ ^,^ ^c^ ^,^ ^e^ ^,^ ^h^ ^,^ ^f^ ^,^ ^g^	4 wk	High
													- I Phase: 10 g thrice a d×48 h				
													- M phase: 10 g OD up to 2 wk				
14	Kang J	2024	China	62	50±12	4.0±0.4	- (HD patients)	100	NA	NA	NA	NA	SZC - 10 g before parathyroidectomy	SOC	^b^ ^,^ ^c^ ^,^ ^f^	1 d	Low
15	Middleton JP	2024	USA	31	56±11	5.7±0.6	- (HD patients)	100	32	16	10	NA	Patiromer - 8.4–25.2 g/d	SOC	b,c,i,e,f	4 wk	Some concerns
16	Charytan DM	2025	USA	88	57±12	5.5±0.4	- (HD patients)	100	55	NA	NA	NA	SZC - 5–15 g/d (non-HD days; 4 times/wk) with dialysate K 3.0 mmol/L	Dialysate K 2.0 mmol/L	^f^ ^,^ ^i^ ^,^ ^j^	8 wk	Low
17	Elsayed MM	2025	Egypt	120	50±12	5.6±0.3	- (HD patients)	100	24	NA	10	0	SZC - 5 g 3 times/wk (15 g/wk)	SPS 15 g 3 times/wk (45 g/wk)	^b^ ^,^ ^f^ ^,^ ^g^	8 wk	Low
18	Kashihara N	2025	Japan	67	69±9	5.8±0.2	NA	100	56	19	78	NA	Patiromer - 8.4–25.2 g/d	Placebo	^b^ ^,^ ^c^ ^,^ ^d^ ^,^ ^e^ ^,^ ^f^ ^,^ ^g^	4 wk	Low
19	Kashihara N	2025	Japan	185	68±9	5.4±0.4	- (Mixed HD/NDD patients)	100	58	12	78	NA	Patiromer - 8.4–25.2 g/d	Placebo	^b^ ^,^ ^d^ ^,^ ^f^ ^,^ ^g^	53 wk	Low

AMBER, Patiromer Versus Placebo to Enable Spironolactone Use in Patients With Resistant Hypertension and Chronic Kidney Disease: a Phase 2, Randomised, Double-Blind, Placebo-Controlled Trial; DIALYZE, DIALIZE China: A Phase IIIb, Randomized, Placebo-Controlled Study to Reduce Predialysis Hyperkalemia With Sodium Zirconium Cyclosilicate in Chinese Patients; DIAMOND, Patiromer for the Management of Hyperkalemia in Heart Failure With Reduced Ejection Fraction; DM, diabetes mellitus; ENERGIZE, Emergency Potassium Normalization Treatment Including Sodium Zirconium Cyclosilicate: A Phase II, Randomized, Double-blind, Placebo-controlled Study; HARMONIZE, The Hyperkalemia Randomized Intervention Multidose ZS-9 Maintenance; HD, hemodialysis; I phase, initial phase; M phase, maintenance phase; NEUTRALIZE, Sodium Zirconium Cyclosilicate in CKD, Hyperkalemia, and Metabolic Acidosis: NEUTRALIZE Randomized Study; NDD, nondialysis dependent; PEARL-HF, Evaluation of the Efficacy and Safety of RLY5016, a Polymeric Potassium Binder, in a Double-Blind, Placebo-Controlled Study in Patients With Chronic Heart Failure; RAAS, renin-angiotensin-aldosterone system; SPS, sodium polystyrene sulfonate; SOC, Standard of care; SZC, sodium zirconium cyclosilicate.

a Outcomes of interest

b Serum potassium reduction.

c Incidence of hyperkalemia.

d Renin-angiotensin-aldosterone system inhibitors optimization (reaching target dose or discontinuing drug).

e Mortality.

f Safety outcome.

g Serum potassium normalization or responder.

h Serum bicarbonate reduction.

i Cardiac arrhythmia.

j Serum potassium outside optimal window.

### Drug Intervention and Phase of Intervention

Drug interventions were classified into two primary categories: (*1*) patiromer (seven studies) and (*2*) SZC (12 studies). Control group interventions consisted of placebo, standard of care, or sodium polystyrene sulfonate (SPS; Table 1). In SZC studies, treatment typically included an initial phase (within the first 48–72 hours) followed by a maintenance phase. The median duration of the maintenance phase was 4 weeks (range, 0.14–8 weeks) for SZC and 4 weeks (range, 4–53 weeks) for patiromer. The treatment duration ranged from 1 day to 53 weeks, with study-specific dosage and frequency (Table 1).

### Risk of Bias Assessment

**Figure 3:**
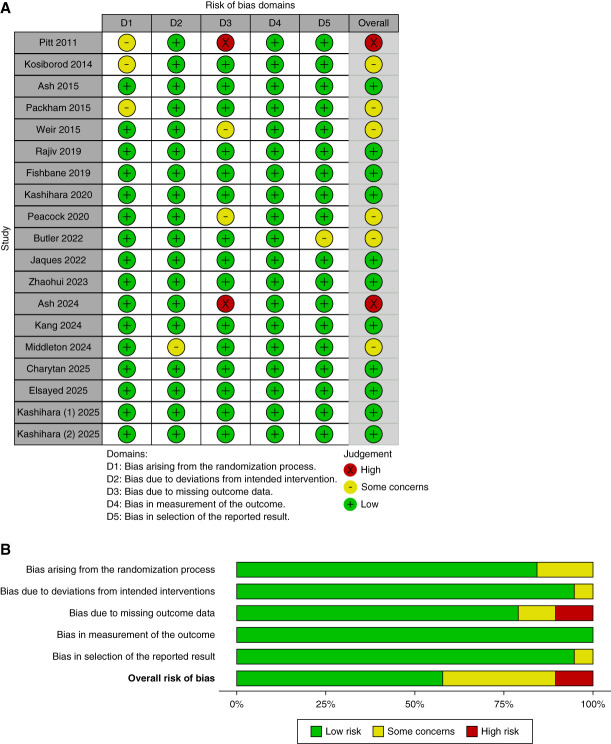
**Risk of bias assessment of included RCTs**. (A) Traffic light plot (B) weighted summary plot of the overall type of bias. RCTs, randomized controlled trials.

Using RoB2, 11 studies were low risk, six had some concerns, and two were high risk of bias. Figure 3 shows RoB2 domain ratings and overall evaluations of included studies. The overall assessment indicated a low to moderate bias (Figure 3, A and B).

### Efficacy of SZC on Laboratory and Clinical Outcomes

#### Effect of SZC on Serum Potassium Levels

##### Initial Phase

**Figure 4:**
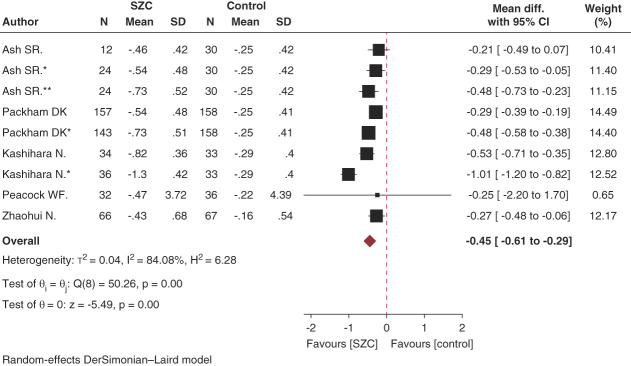
**Forest plot displaying the mean difference in serum potassium reduction between SZC and control at the end of initial phase.** The study by Ash.^19^ *et al*. were subdivided by SZC dosage groups—0.3, 3, and 10 g—administered once daily and represented as Ash SR., Ash SR.*, and Ash SR.**, respectively. The study by Packham.^20^ *et al*. was divided into 5 and 10 g three-times-daily SZC groups, labeled as Packham DK. and Packham DK.*, respectively. The study by Kashihara.^22^ *et al*. were divided into 5 and 10 g SZC groups administered three times daily, labeled as Kashihara N. and Kashihara N.*, respectively. CI, confidence interval.

As presented in Figure 4 and Table 2, SZC reduced serum potassium levels at the end of the initial phase compared with the controls (WMD: −0.45 mEq/L [95% CI, −0.61 to −0.29]; *P* < 0.01). The results were consistent across all subgroups, including the percentage of patients with CKD, DM, or those using RAAS inhibitors, dialysis status, risk of bias, and race (Supplemental Table 2).

**Table 2:** Summary of the efficacy outcomes of sodium zirconium cyclosilicate treatment

Outcomes	No. of Studies	No. of Patients			Effect Estimate (95% CI)	*I*^2^ (%)
		Total	SZC	Control		
**Serum potassiumreduction (mEq/L)**						
Initiation phase						
*At 1 h*	7	452	224	228	WMD −0.13 (−0.21 to −0.04)^a^	0
*At 2 h*	7	452	224	228	WMD −0.04 (−0.12 to 0.04)	0
*At 4 h*	7	452	224	228	WMD −0.07 (−0.16 to 0.02)	0
*At 24 h*	6	384	192	192	WMD −0.28 (−0.37 to −0.19)^a^	15.3
*At 48–72 h*	5	885	436	449	WMD −0.51 (−0.73 to −0.29)^a^	91.3
*End of initial*	9	1103	528	572	WMD −0.45 (−0.61 to −0.29)^a^	84.1
Maintenance phase						
*At 4 wk*	5	565	232	333	WMD −0.61 (−0.75 to −0.48)^a^	38.2
*End of maintenance*	7	725	325	400	WMD −0.50 (−0.63 to −0.38)^a^	67.5
Serum HCO_3_^−^ improvement (mEq/L)						
*End of study*	5	633	397	236	WMD+0.56 (0.12 to 1.01)^a^	16.1
Serum potassium and clinical outcomes						
*Responders*	8	1663	809	854	RR 1.49 (1.17 to 1.89)^a^	81.2
*Incidence of hyperkalemia*	11	1911	885	1026	RR 0.37 (0.23 to 0.58)^a^	84.7
*Serum potassium outside optimal window*	6	837	372	465	RR 0.29 (0.18 to 0.46)^a^	68.1
*Mortality*	10	985	421	564	RR 1.33 (0.43 to 4.10)	0
*Arrhythmia*	3	308	156	152	RR 0.90 (0.44 to 1.84)	0

CI, confidence interval; RR, relative risk; SZC, sodium zirconium cyclosilicate; WMD, weighted mean difference.

a Results were statistically significant with a *P* value <0.05.

In the initial phase of treatment, SZC reduced serum potassium levels compared with the controls over time: at 1 hour (WMD: −0.13 mEq/L [95% CI, −0.21 to −0.04]; *P* < 0.01), 24 hours (WMD: −0.28 mEq/L [95% CI, −0.37 to −0.19]; *P* < 0.01), and 48 hours (WMD: −0.51 mEq/L [95% CI, −0.73 to −0.29]; *P* < 0.01; Table 3). However, SZC did not demonstrate a statistically significant reduction in serum potassium at 2 hours or 4 hours.

**Table 3:** Summary of the efficacy outcomes of patiromer treatment

Outcomes	No. of Studies	No. of Patients			Effect Estimate (95% CI)	*I*^2^ (%)
		Total	Patiromer	Control		
**Serum potassium reduction (mEq/L)**						
Maintenance phase						
*At 4 wk*	3	278	144	134	WMD −0.40 (−0.69 to −0.11)^a^	91.6
*End of maintenance*	7	1454	733	721	WMD −0.36 (−0.51 to −0.21)^a^	92.9
Clinical outcomes						
*Incidence of hyperkalemia*	8	1719	865	854	RR 0.55 (0.40 to 0.76)^a^	77.1
*Reaching target RAAS inhibitors*	4	1331	667	664	RR 1.23 (1.05 to 1.46)^a^	72.3
*Discontinuing RAAS inhibitors*	4	2158	1080	1078	RR 0.43 (0.23 to 0.80)^a^	71.4
*Mortality*	6	1471	739	732	RR 1.22 (0.68 to 2.18)	0
*Arrhythmia*	2	138	71	67	RR 0.88 (0.36 to 2.14)	0

CI, confidence interval; RAAS, renin angiotensin aldosterone system; RR, relative risk; WMD, weighted mean difference.

a Results were statistically significant with a *P* value <0.05.

##### Maintenance Phase

**Figure 5:**
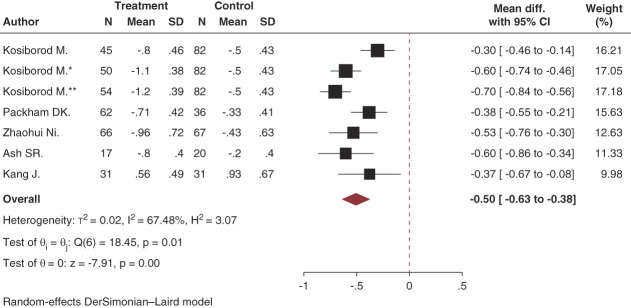
**Forest plot displaying the mean difference in serum potassium reduction between SZC and control at the end of the study.** The studies by Kosiborod.^18^ *et al*. were subdivided by SZC dosage groups—5, 10, and 15 g—administered once daily and represented as Kosiborod M., Kosiborod M.*, and Kosiborod M.**, respectively.

As presented in Figure 5 and Table 2, SZC reduced serum potassium levels at the end of the maintenance phase compared with the controls (WMD, −0.50 mEq/L; 95% CI, −0.63 to −0.38; *P* < 0.01). Effects were consistent across dialysis status, risk of bias, and race (Supplemental Table 3). At 4 weeks, SZC also reduced serum potassium (WMD: −0.61 mEq/L; 95% CI, −0.75 to −0.48; *P* < 0.01).

#### Effect of SZC on Serum Bicarbonate Levels

As presented in Table 2, SZC increased serum bicarbonate levels at the end of the study compared with the controls (WMD, +0.56 mEq/L; 95% CI, 0.12 to 1.01; *P* = 0.01).

#### Effect of SZC on Serum Potassium and Clinical Outcomes

##### Proportion of Responders

Five articles,^20,21,23–25^ comprising eight study arms based on differing SZC dosing regimens, reported the proportion of responders, as defined in each individual study (Supplemental Table 4) in 1663 patients. SZC increased likelihood of maintaining normokalemia compared with the controls (RR, 1.49; 95% CI, 1.17 to 1.89; *P* < 0.01; Table 2).

##### Incidence of Hyperkalemia

Six articles,^18,20,23,25,26,28^ encompassing 11 study arms stratified by different SZC dosing regimens, evaluated the incidence of hyperkalemia, with definitions specified in each study (Supplemental Table 5) in 1911 patients. SZC significantly reduced hyperkalemia risk compared with the controls (RR, 0.37; 95% CI, 0.23 to 0.58; *P* < 0.01; Table 2).

##### Risk of serum potassium outside the optimal range

Four articles,^18,22,24,27^ comprising six study arms based on varying SZC dosing strategies, assessed the risk of serum potassium levels falling outside the optimal range, with definitions specified in each study (Supplemental Table 6), in 837 patients. SZC reduced risk of serum potassium outside the optimal range compared with the controls (RR, 0.29; 95% CI, 0.18 to 0.46; *P* < 0.01; Table 2).

#### Mortality

Six articles,^18,19,21,23–25^ encompassing ten study arms with different SZC dosing regimens, reported mortality outcomes in 837 patients. There was no significant difference in mortality between the SZC and control groups (Table 2).

#### Cardiac Arrhythmia

Two articles,^22,27^ encompassing three study arms with different SZC dosing regimens, reported cardiac arrhythmia outcomes among 308 patients. There was no significant difference in cardiac arrhythmia between the SZC and control groups (Table 2).

### Efficacy of Patiromer on Serum Potassium Reduction and Clinical Outcomes

#### Effect of Patiromer on Serum Potassium Levels

**Figure 6:**
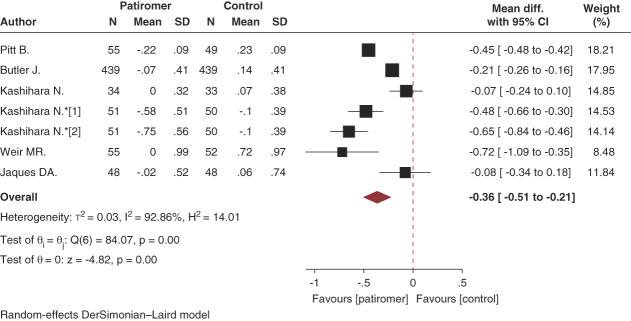
**Forest plot displaying the mean difference in serum potassium reduction between patiromer and control.** The study by Kashihara N. corresponds to ref. ^16^, while Kashihara N.* refer to ref. ^17^. The study by Kashihara N.* *et al*. were subdivided by patiromer dosage groups—8.4, and 16.8—administered once daily and represented as Kashihara N.*,^1^ and Kashihara N.**,^2^ respectively.

Figure 6 and Table 3 present that patiromer reduced serum potassium levels at the end of the maintenance phase compared with the controls (WMD, −0.36 mEq/L; 95% CI, −0.51 to −0.21; *P* < 0.01). Subgroup analyses (Supplemental Table 7) demonstrated consistent efficacy across studies regardless of risk of bias and race (*P* for interaction=0.70 and 0.26, respectively). No significant differences were observed between subgroups with more or <50% of patients having DM or heart failure. Studies with >50% of participants had CKD or were receiving RAAS inhibitors demonstrated a significantly greater reduction in serum potassium with patiromer compared with studies with lower proportions (<50%) of these populations (*P* for interaction=0.04 and 0.01, respectively). Among CKD patients, those with non–dialysis-dependent CKD experienced a significantly greater reduction in serum potassium with patiromer compared with those undergoing dialysis (*P* for interaction=0.04). At 4 weeks, patiromer reduced serum potassium (WMD: −0.40 mEq/L; 95% CI, −0.69 to −0.11; *P* < 0.01; Table 3).

#### Effect of Patiromer on Serum Potassium and Clinical Outcomes

##### Incidence of Hyperkalemia

Seven articles^10–15,17^ involving eight studies with different patiromer dosage regimens in a total of 1719 patients assessed the incidence of hyperkalemia. Hyperkalemia definitions were based on criteria outlined in each individual study (Supplemental Table 5). Patiromer reduced hyperkalemia risk compared with the controls (RR, 0.55; 95% CI, 0.40 to 0.76; *P* < 0.01; Table 3).

##### Proportion of Patients Achieving Target Dose of RAAS Inhibitors

Four studies,^10,12,13,16^ including a total of 1331 patients, evaluated the proportion of individuals who achieved the target dose of RAAS inhibitors. Three studies^10,12,13^ assessed this outcome during the dose-titration phase, while one study from Kashihara *et al*.^16^ evaluated it during the maintenance phase at the target dose. Regarding the types of RAAS inhibitors examined, Pitt *et al*.^10^ and Agarwal *et al*.^12^ focused specifically on spironolactone, whereas Butler *et al*.^13^ and Kashihara *et al*.^16^ evaluated a broader range of RAAS inhibitors, including angiotensin-converting enzyme inhibitors, angiotensin receptor blockers, angiotensin receptor-neprilysin inhibitors, and mineralocorticoid receptor antagonist, each using study-specific titration protocols. Patiromer increased likelihood of reaching the target RAAS inhibitors dose compared with the controls (RR, 1.23; 95% CI, 1.05 to 1.46; *P* = 0.01; Table 3).

##### Proportion of Patients Discontinuing RAAS Inhibitors Due to Hyperkalemia

Three articles,^11–13^ involving four studies with different patiromer dosage regimens in 2158 patients, assessed the discontinuation rate of RAAS inhibitors due to hyperkalemia. Two articles^12,13^ evaluated this outcome during the dose-titration phase, whereas one article by Weir *et al*.^11^ assessed it during the maintenance phase at the target dose. With respect to the RAAS inhibitor classes examined, Agarwal *et al*.^12^ focused exclusively on spironolactone, while Butler *et al*.^13^ conducted separate analyses for mineralocorticoid receptor antagonists (spironolactone and eplerenone) and other RAAS inhibitors, including angiotensin-converting enzyme inhibitors, angiotensin receptor blockers, and angiotensin receptor-neprilysin inhibitors. By contrast, Weir *et al*.^11^ assessed discontinuation across RAAS inhibitors without specifying individual classes. Patiromer reduced likelihood of RAAS inhibitors discontinuation compared the controls (RR, 0.43; 95% CI, 0.23 to 0.80; *P* = 0.01; Table 3).

##### Mortality

Six studies,^10,12–16^ comprising 1471 patients, reported mortality outcome. There was no significant difference in all-cause mortality between the patiromer and control groups (Table 3).

##### Cardiac Arrhythmia

Two studies,^11,15^ involving 138 patients, evaluated the incidence of cardiac arrhythmia. There was no significant difference in arrhythmic events between patiromer and control groups (Table 3).

### Tolerability and Safety Profiles of SZC and Patiromer

#### Adverse Drug Events of SZC

As summarized in Table 4, no statistically significant differences were observed between the SZC and control groups in the incidence of overall adverse events, serious adverse events, nausea, vomiting, diarrhea, constipation, or hypokalemia. However, SZC was associated with a significantly higher risk of edema compared with control. One study^18^ reported that most patients who developed SZC-associated edema (13 of 14; 93%) were able to complete the study, with 50% requiring no treatment modification, whereas another study^26^ reported no cases of edema.

**Table 4:** Summary of the safety and tolerability outcomes of sodium zirconium cyclosilicate and patiromer treatment

Adverse Drug Events	No. of Studies	No. of Patients	Relative Risk (95% CI)	*P*-Value	Assessment of Heterogeneity		Egger's Test *P* Value
					*I*^2^ (%)	*P* Value	
**SZC**							
Any AE.	10	2355	1.11 (0.99 to 1.25)	0.08	7.5	0.36	0.25
Serious AE.	6	920	1.26 (0.69 to 2.30)	0.46	26.4	0.22	0.08
Nausea	5	949	1.51 (0.65 to 3.51)	0.33	0	0.70	0.11
Vomiting	2	634	2.65 (0.98 to 7.15)	0.06	0	0.71	0.08
Diarrhea	5	1083	0.98 (0.51 to 1.86)	0.94	0	0.76	0.83
Constipation	7	1423	1.19 (0.63 to 2.28)	0.59	1.7	0.43	0.008
Edema	2	299	2.92 (1.08 to 7.90)^a^	0.04^a^	0^a^	0.55^a^	0.26
Hypokalemia	10	1685	1.38 (0.71 to 2.68)	0.34	20.1	0.23	0.001
**Patiromer**							
Any AE.	6	1482	1.06 (0.92 to 1.22)	0.40	29.5	0.21	0.055
Serious AE.	6	1547	0.96 (0.69 to 1.34)	0.83	0	0.77	0.62
Nausea	2	985	1.31 (0.37 to 4.59)	0.67	0	0.36	0.45
Diarrhea	4	1347	1.23 (0.73 to 2.07)	0.44	0	0.57	0.26
Constipation	4	1156	2.96 (1.21 to 7.22)^a^	0.02^a^	0^a^	0.76^a^	0.30
Hypokalemia	5	1451	1.46 (1.05 to 2.03)^a^	0.03^a^	0^a^	0.85^a^	0.45
Hypomagnesemia	4	1156	1.81 (0.40 to 8.15)	0.44	49.7	0.11	0.96

AE, adverse event; CI, confidence interval; SZC, Sodium zirconium cyclosilicate.

a Results were statistically significant with a *P* value <0.05.

#### Adverse Drug Events of Patiromer

Table 4 summarizes adverse events of patiromer. No significant differences were observed between the patiromer and control groups in the incidence of total adverse events, serious adverse events, hypomagnesemia, or GI symptoms such as nausea and diarrhea. However, patiromer increased the risk of constipation and hypokalemia compared with the controls. Among studies evaluating the safety of patiromer, hypokalemia was reported as mild in two of the five studies,^13,16^ while the remaining studies^10–12^ did not report severity. Constipation was described as mild to moderate in three of the four studies,^10,11,16^ whereas Butler *et al*.^13^ did not specify the severity of this adverse event.

### Meta-Regression

Meta-regression of outcomes with greater than ten studies (serum potassium reduction with SZC at end of initial and maintenance phases, and with patiromer at end of maintenance) showed no association between treatment effect and age, sample size, baseline serum potassium, or baseline GFR (Supplemental Table 8).

### Assessment of Sensitivity Analysis and Publication Bias

Leave-one-out sensitivity analysis showed that heterogeneity did not change significantly for efficacy outcomes. Publication bias was assessed through visual inspection of funnel plots and statistical evaluation using the Egger’s regression test. The funnel plots corresponding to the efficacy of SZC in reducing serum potassium during both the initial and maintenance phases appeared symmetrical (Supplemental Figures 1 and 2), indicating no apparent publication bias. This observation was supported by the Egger’s test, which yielded nonsignificant results. Similarly, for patiromer, the funnel plot assessing serum potassium reduction during the maintenance phase was also symmetrical (Supplemental Figure 3), with the Egger’s test indicating no significant publication bias (*P* = 0.50).

## Discussion

Our systematic review evaluated the efficacy and tolerability of SZC and patiromer in the management of hyperkalemia. Meta-analyses of 19 randomized controlled trials involving 3627 patients demonstrated that, during the initial phase, a significant reduction in serum potassium levels was observed in patients treated with SZC. Data on patiromer during the initial phase are lacking, likely due to differences in study design, dosing regimen and its inherently slower onset of action, which is primarily intended for managing chronic hyperkalemia. During the maintenance phase, both SZC and patiromer were associated with significantly lower serum potassium levels compared with control groups. Furthermore, an improvement in metabolic acidosis was observed at the end of the maintenance phase among patients receiving SZC. SZC appeared effective in normalizing and sustaining serum potassium levels while reducing the incidence of hyperkalemia during therapy. Patiromer demonstrated efficacy in enabling patients with hyperkalemia—or those at risk—to achieve target doses of RAAS inhibitors while also reducing the rate of RAAS inhibitors discontinuation related to hyperkalemia. However, neither therapy showed a significant impact on mortality rates or the incidence of cardiac arrhythmias, possibly due to limited statistical power and insufficient duration of follow-up. Regarding safety and tolerability, neither SZC nor patiromer was associated with a significant increase in the overall incidence of adverse events or serious adverse events compared with control groups. However, SZC was linked to a higher incidence of edema, while patiromer was associated with increased rates of constipation and hypokalemia.

Consistent with previous meta-analyses,^29,30^ our analysis in a more diverse population confirms that both SZC and patiromer effectively reduce serum potassium levels during the maintenance phase of chronic hyperkalemia management (Figures 5 and 6). Reflecting their clinical efficacy, the Kidney Disease Improving Global Outcomes 2024 Clinical Practice Guidelines for CKD^31^ recommend these two licensed potassium-binding agents as second-line treatment options, following the correction of reversible causes of chronic hyperkalemia—such as the withdrawal of non–RAAS inhibitors medications and the avoidance of high-potassium processed foods. Their effective potassium-lowering action can lead to clinically meaningful outcomes by reducing the risk of deviations in serum potassium levels from the optimal range in both non–dialysis-dependent CKD and dialysis-dependent CKD patients (Supplemental Table 3), as well as by facilitating the achievement of target RAAS inhibitors dosing (Table 3). According to Epstein *et al*., among 205,108 patients, those with comorbid CKD, DM, and heart failure who received suboptimal doses of RAAS inhibitors or discontinued treatment were at significantly higher risk of cardio-renal adverse outcomes and increased mortality compared with those receiving maximal dosing.^32^ Patiromer proved beneficial in allowing patients to achieve targeted RAAS inhibitors doses and in decreasing treatment interruptions due to hyperkalemia (Table 4). In subgroup analyses, studies enrolling a higher proportion of participants with CKD or those treated with RAAS inhibitors (>50%) demonstrated a significantly greater reduction in serum potassium with patiromer compared with those with lower proportions (<50%; Supplemental Table 7). This observation underscores the therapeutic value of patiromer in patients at heightened risk of hyperkalemia, supporting its role in facilitating continued RAAS inhibitors use in CKD.

In patients with kidney failure receiving hemodialysis, the capacity to maintain potassium balance is markedly reduced. Sudden shifts in extracellular potassium concentration and the resulting changes in membrane potential can precipitate life-threatening arrhythmias.^33^ Our analysis demonstrated that administering SZC on nondialysis days significantly reduced predialysis serum potassium (Supplemental Tables 2 and 3) and that both potassium binders significantly reduced the incidence of hyperkalemia during treatment—regardless of dialysis status. These findings suggest that patients with dialysis-dependent CKD treated with either of these novel potassium binders may serve as adjuncts in facilitating a more liberal, potassium-rich, high-fiber, plant-based diet—an approach associated with cardiovascular and health-related quality-of-life benefits.^34,35^ Although the observed reduction in cardiac arrhythmia incidence did not reach statistical significance (Tables 2 and 3), longer follow-up periods and larger sample sizes are required to assess this outcome more definitively.

In addition, we further examined that SZC exclusively provides a rapid onset of action in lowering serum potassium levels within 1 hour (Table 2). This rapid effect may be attributed to SZC's highly selective, high-capacity crystalline lattice structure, which allows it to bind potassium early in the upper GI tract—where potassium is abundant but less concentrated^36^—consistent with findings from an *in vitro* pharmacodynamic study.^37^ Unlike SPS and patiromer, which act mainly in the colon, SZC appears to have a broader activity range, including the duodenum and jejunum.^38^ In light of these mechanisms, Marshall *et al*.^39^ retrospectively evaluated the use of SZC for the emergency management of hyperkalemia and observed a significant decrease in the need for emergency hemodialysis by 77% and the use of temporary central venous catheters by 73%. However, several important limitations should be noted in this real-world evidence study, including the inability to fully account for unmeasured confounders. Following the Emergency Potassium Normalization Treatment Including Sodium Zirconium Cyclosilicate: A Phase 2, Randomized, Double-blind, Placebo-controlled study^23^ demonstrating the incremental advantage of adding SZC to insulin and glucose in the emergency treatment of hyperkalemia, the National Institute for Health and Care Excellence^40^ approved SZC as a complementary therapy alongside conventional treatments for acute hyperkalemia. Our analysis also highlighted an additional metabolic benefit of SZC—its capacity to improve metabolic acidosis (Table 2)—a benefit that may be particularly relevant in patients with CKD. This effect likely occurs by binding ammonium ions and reducing their intestinal absorption, thereby decreasing hepatic urea recycling and overall acid load, rather than through direct hydrogen ion excretion *via* the feces.^41^

Our analysis highlights key safety considerations for the use of SZC and patiromer in managing hyperkalemia. Both agents appear to be well-tolerated potassium binders, with no significant increase in the incidence of adverse or serious adverse events compared with control groups (Table 4). By contrast, traditional potassium exchange resins such as SPS and calcium polystyrene sulfonate have raised concerns about the potential risk of severe GI complications, particularly intestinal necrosis and perforation.^42,43^ Notably, no cases of such severe GI disorders associated with SZC or patiromer were reported in the included studies, which is consistent with findings from postmarketing and global pharmacovigilance data reported by Chen *et al*.^44^ and Rossignol *et al*.^45^ Nonetheless, some concerns regarding edema were reported more frequently in patients treated with SZC (RR, 2.92; *P* = 0.04; Table 4). This adverse effect is likely related to the sodium exchange properties of the compound, given that each 5 g dose contributes approximately 400 mg of sodium, corresponding to around 20% of the World Health Organization's recommended upper limit for daily sodium intake.^46^ This emphasizes the need for careful monitoring of volume status, particularly in patients with heart failure or advanced CKD. Notably, patiromer was associated with higher rates of constipation (Table 4). The increased incidence of constipation aligns with findings from global pharmacovigilance data^44^ and is generally of mild to moderate severity. However, caution is warranted in patients with impaired bowel motility, such as those with ileus.

This study has several strengths. It provides a comprehensive review evaluating the efficacy of SZC and patiromer across various patient comorbidities at risk for hyperkalemia. In addition, by assessing the tolerability profiles of both agents, this multiobjective analysis was designed to support clinical decision-making and identify the optimal intervention for these patient populations. Subgroup analyses and meta-regression were also conducted to identify key moderators influencing the relationship between these potassium binders and the outcomes of interest. Nevertheless, our analysis is subject to some important limitations. First, significant heterogeneity was observed across some outcomes, particularly regarding efficacy for both potassium binders. This heterogeneity likely reflects variations in patient characteristics; definitions of hyperkalemia, treatment response, and optimal range of serum potassium; baseline serum potassium and renal function, as well as differences in drug dosage and duration of therapy among included studies, which may have introduced uncertainty into the pooled effect estimates. Second, the follow-up duration for cardiac arrhythmia and mortality outcomes was relatively short, as previously noted, and the limited sample size and number of studies may have limited the ability to detect significant effects. Finally, the mean difference in serum potassium reduction may not reflect the true effect of the potassium binders alone, as several studies compared these agents with nonplacebo control groups receiving standard care.

Both SZC and patiromer effectively lower serum potassium and provide clinical benefits, with distinct safety considerations. Therapy selection should be guided by treatment goals and the patient's individual risk profile. Future research should further explore patient-centered outcomes such as quality of life, dietary freedom, and health care cost implications.

## Supplementary Material

## Data Availability

All original data, including deidentified patient-level data or individual laboratory data measurements, are included in the manuscript and/or supplemental material.

## Supplemental Material

Supplemental Table 1. Keywords for article searches.

Supplemental Table 2. Subgroup analyses examining the effect of SZC on serum potassium reduction at the end of initial phase.

Supplemental Table 3. Subgroup analyses examining the effect of SZC on serum potassium reduction at the end of maintenance phase.

Supplemental Table 4. Definitions of responders.

Supplemental Table 5. Definitions of hyperkalemia used across included studies.

Supplemental Table 6. Definitions of the optimal range of serum potassium utilized across included studies.

Supplemental Table 7. Subgroup analyses examining the effect of patiromer on serum potassium reduction at the end of study.

Supplemental Table 8. Results of meta-regression between variables and serum potassium reduction from SZC and patiromer.

Supplemental Figure 1. Funnel plot displaying the standard error by the mean difference in serum potassium reduction following the initial phase of SZC treatment across individual studies. (*P* = 0.77).

Supplemental Figure 2. Funnel plot displaying the standard error by the mean difference in serum potassium reduction following the maintenance phase of SZC treatment across individual studies (*P* = 0.68).

Supplemental Figure 3. Funnel plot displaying the standard error by the mean difference in serum potassium reduction following the maintenance phase of patiromer treatment across individual studies (*P* = 0.50).
