# Supplementary material for: Efficacy and Tolerability of Sodium Zirconium Cyclosilicate or Patiromer in Managing Hyperkalemia: A Systematic Review and Meta-Analysis
Source: Kidney360. 2026 Mar 11;7(7):1601–14. doi: 10.34067/KID.0000001173 (PMC13450949; doi:10.34067/KID.0000001173)
Supplement: Supplementary file 1 [file kidney360-7-1601-s001.pdf]

ASN American Society of Nephrology
JASN Journal of the American Society of Nephrology
KIDNEY360 Accessing Our World From Every Angle
CJASN Clinical Journal of the American Society of Nephrology

ASN Journal Disclosure Form

As per ASN journal policy, I have disclosed any financial relationships or commitments I have held in the past 36 months as included below. I have listed my Current Employer below to indicate there is a relationship requiring disclosure. If no relationship exists, my Current Employer is not listed.

W. Cheungpasitporn reports the following:
Employer: Mayo Clinic, Rochester, Minnesota

I understand that the information above will be published within the journal article, if accepted, and that failure to comply and/or to accurately and completely report the potential financial conflicts of interest could lead to the following: 1) Prior to publication, article rejection, or 2) Post-publication, sanctions ranging from, but not limited to, issuing a correction, reporting the inaccurate information to the authors' institution, banning authors from submitting work to ASN journals for varying lengths of time, and/or retraction of the published work.

Name: Wisit Cheungpasitporn
Manuscript ID: K360-2025-001494R1
Manuscript Title: Efficacy and Tolerability of Sodium Zirconium Cyclosilicate and Patiromer in Managing Hyperkalemia: A Systematic Review and Meta-Analysis
Date of Completion: January 23, 2026
Disclosure Updated Date: January 14, 2026

ASN American Society of Nephrology JASN Journal of the American Society of Nephrology KIDNEY360 Accessing Our World From Every Angle CJASN Clinical Journal of the American Society of Nephrology

ASN Journal Disclosure Form

As per ASN journal policy, I have disclosed any financial relationships or commitments I have held in the past 36 months as included below. I have listed my Current Employer below to indicate there is a relationship requiring disclosure. If no relationship exists, my Current Employer is not listed.

S. Jaturapisanukul reports the following:
Employer: Vajira Hospital; and Honoraria: Boehringer company; Pfizer; AstraZeneca.

I understand that the information above will be published within the journal article, if accepted, and that failure to comply and/or to accurately and completely report the potential financial conflicts of interest could lead to the following: 1) Prior to publication, article rejection, or 2) Post-publication, sanctions ranging from, but not limited to, issuing a correction, reporting the inaccurate information to the authors' institution, banning authors from submitting work to ASN journals for varying lengths of time, and/or retraction of the published work.

Name: Solos Jaturapisanukul
Manuscript ID: K360-2025-001494R1
Manuscript Title: Efficacy and Tolerability of Sodium Zirconium Cyclosilicate and Patiromer in Managing Hyperkalemia: A Systematic Review and Meta-Analysis
Date of Completion: January 23, 2026
Disclosure Updated Date: January 23, 2026

ASN Journal Disclosure Form

As per ASN journal policy, I have disclosed any financial relationships or commitments I have held in the past 36 months as included below. I have listed my Current Employer below to indicate there is a relationship requiring disclosure. If no relationship exists, my Current Employer is not listed.

C. Thongprayoon reports the following:
Employer: Mayo Clinic; and Ownership Interest: Apple; Meta; Microsoft; Google; AT&T; Verizon; ZIM; GlaxosmithKline.

I understand that the information above will be published within the journal article, if accepted, and that failure to comply and/or to accurately and completely report the potential financial conflicts of interest could lead to the following: 1) Prior to publication, article rejection, or 2) Post-publication, sanctions ranging from, but not limited to, issuing a correction, reporting the inaccurate information to the authors' institution, banning authors from submitting work to ASN journals for varying lengths of time, and/or retraction of the published work.

Name: Charat Thongprayoon
Manuscript ID: K360-2025-001494R1
Manuscript Title: Efficacy and Tolerability of Sodium Zirconium Cyclosilicate and Patiromer in Managing Hyperkalemia: A Systematic Review and Meta-Analysis
Date of Completion: January 22, 2026
Disclosure Updated Date: January 14, 2026

ASN American Society of Nephrology | JASN Journal of the American Society of Nephrology | KIDNEY360 Accessing Our World From Every Angle | CJASN Clinical Journal of the American Society of Nephrology

ASN Journal Disclosure Form

As per ASN journal policy, I have disclosed any financial relationships or commitments I have held in the past 36 months as included below. I have listed my Current Employer below to indicate there is a relationship requiring disclosure. If no relationship exists, my Current Employer is not listed.

W. Wathanavasin has nothing to disclose.

I understand that the information above will be published within the journal article, if accepted, and that failure to comply and/or to accurately and completely report the potential financial conflicts of interest could lead to the following: 1) Prior to publication, article rejection, or 2) Post-publication, sanctions ranging from, but not limited to, issuing a correction, reporting the inaccurate information to the authors' institution, banning authors from submitting work to ASN journals for varying lengths of time, and/or retraction of the published work.

Name: Wannasit Wathanavasin
Manuscript ID: K360-2025-001494R1
Manuscript Title: Efficacy and Tolerability of Sodium Zirconium Cyclosilicate and Patiromer in Managing Hyperkalemia: A Systematic Review and Meta-Analysis
Date of Completion: January 23, 2026
Disclosure Updated Date: January 23, 2026
