# Supplementary material for: Efficacy and Tolerability of Sodium Zirconium Cyclosilicate or Patiromer in Managing Hyperkalemia: A Systematic Review and Meta-Analysis
Source: Kidney360. 2026 Mar 11;7(7):1601–14. doi: 10.34067/KID.0000001173 (PMC13450949; doi:10.34067/KID.0000001173)
Supplement: Supplementary file 2 [file kidney360-7-1601-s002.pdf]

Supplemental Table 1. Keywords for article searches

Keywords	No. of articles
Pubmed	
"sodium zirconium cyclosilicate"[Supplemental Concept] OR "sodium zirconium cyclosilicate"[All Fields] OR "SZC"[All Fields] OR "ZS-9"[All Fields] OR ("patiromer"[Supplemental Concept] OR "patiromer"[All Fields]) OR ("patiromer"[Supplemental Concept] OR "patiromer"[All Fields] OR "rly5016"[All Fields])) AND ("hyperkalaemia"[All Fields] OR "hyperkalemia"[MeSH Terms] OR "hyperkalemia"[All Fields] OR "hyperkalemias"[All Fields] OR (("serum"[MeSH Terms] OR "serum"[All Fields] OR "serums"[All Fields] OR "serum s"[All Fields] OR "serumal"[All Fields]) AND ("potassium dietary"[Supplemental Concept] OR "potassium dietary"[All Fields] OR "potassium"[All Fields] OR "potassium"[Supplemental Concept] OR "potassium, dietary"[MeSH Terms] OR ("potassium"[All Fields] AND "dietary"[All Fields]) OR "dietary potassium"[All Fields] OR "potassium"[MeSH Terms])) OR (("potassium dietary"[Supplemental Concept] OR "potassium dietary"[All Fields] OR "potassium"[All Fields] OR "potassium"[Supplemental Concept] OR "potassium, dietary"[MeSH Terms] OR ("potassium"[All Fields] AND "dietary"[All Fields]) OR "dietary potassium"[All Fields] OR "potassium"[MeSH Terms]) AND ("reduction"[All Fields] OR "reductions"[All Fields])) OR "hyperkalemia"[MeSH Terms] OR ("potassium, dietary"[MeSH Terms] OR "potassium"[MeSH Terms]	**428**
Scopus	
((TITLE-ABS-KEY ("hyperkalemia") OR TITLE-ABS-KEY ("serum potassium") OR TITLE-ABS-KEY ("potassium reduction"))) AND ((TITLE-ABS-KEY ("patiromer") OR TITLE-ABS-KEY ("RLY5016") OR TITLE-ABS-KEY ("sodium zirconium cyclosilicate") OR TITLE-ABS-KEY ("SZC") OR TITLE-ABS-KEY ("ZS-9")))	**652**
CENTRAL	
"patiromer" OR "RLY5016" OR "sodium zirconium cyclosilicate" OR "SZC" OR "ZS-9" AND "hyperkalemia" OR "serum potassium" OR "potassium reduction"	**272**

Supplemental Table 2. Sub-group analyses examining the effect of SCZ on serum potassium reduction at the end of initial phase.

Subgroup analyses	No. of studies	WMD (95% CI)	P-values	Assessment of heterogeneity		P for interaction
				I^2 index	P-value	
**Participants with CKD**						0.84
<50%	1	-0.25 (-2.20 to 1.70)	0.80	0%	1.00	
≥50%	8	-0.45 (-0.61 to -0.29)	<0.01	86.1%	<0.01	
**Participants with DM**						0.82
<50%	1	-0.25 (-2.20 to 1.70)	0.80	0%	1.00	
≥50%	7	-0.47 (-0.65 to -0.30)	<0.01	87.4%	<0.01	
**Participants with HF**						-
<50%	4	-0.57 (-0.82 to -0.31)	<0.01	93.0%	<0.01	
≥50%	-	-	-	-	-	
**% RAASi use**						0.10
<50%	2	-0.77 (-1.24 to -0.30)	<0.01	92%	<0.01	
≥50%	5	-0.36 (-0.48 to -0.25)	<0.01	56.7%	0.06	
**Dialysis status**						0.15
NDD-CKD	8	-0.47 (-0.65 to -0.30)	<0.01	85.4%	<0.01	
DD-CKD	1	-0.27 (-0.48 to -0.06)	0.01	0%	1.00	
**Risk of bias**						0.57
Low	6	-0.47 (-0.72 to -0.22)	<0.01	87%	<0.01	
Some concern	3	-0.38 (-0.55 to -0.21)	<0.01	70.4%	0.03	
High	-	-	-	-	-	
**Race**						0.27
Asian	3	-0.60 (-1.02 to -0.19)	<0.01	92.7%	<0.01	
Non-Asian	6	-0.36 (-0.47 to -0.26)	<0.01	45.9%	0.10	

Abbreviations: CI, confidence interval; CKD, chronic kidney disease; DD, dialysis-dependent; DM, diabetes mellitus; HF, heart failure; NDD, non–dialysis-dependent; RAASi, renin–angiotensin–aldosterone system inhibitors; RR, relative risk; sK^+, serum potassium; SZC, sodium zirconium cyclosilicate; WMD, weighted mean difference

Supplemental Table 3. Sub-group analyses examining the effect of SCZ on serum potassium reduction at the end of maintenance phase.

Subgroup analyses	No. of studies	WMD (95% CI)	P-values	Assessment of heterogeneity		P for interaction
				I^2 index	P-value	
**Participants with CKD**						-
<50%	7	-0.50 (-0.63 to -0.38)	<0.01	67.5%	0.01	
≥50%	-	-	-	-	-	
**Participants with DM**						-
<50%	-	-	-	-	-	
≥50%	5	-0.51 (-0.67 to -0.36)	<0.01	77.1%	<0.01	
**Participants with HF**						-
<50%	5	-0.51 (-0.67 to -0.36)	<0.01	77.1%	<0.01	
≥50%	-	-	-	-	-	
**% RAASi use**						-
<50%	-	-	-	-	-	
≥50%	4	-0.50 (-0.68 to -0.31)	<0.01	82.5%	<0.01	
**Dialysis status**						0.71
NDD-CKD	5	-0.51 (-0.67 to -0.36)	<0.01	77.1%	<0.01	
DD-CKD	2	-0.47 (-0.65 to -0.29)	<0.01	0%	0.41	
**Risk of bias**						0.72
Low	2	-0.47 (-0.65 to -0.29)	<0.01	0%	0.41	
Some concern	4	-0.50 (-0.68 to -0.31)	<0.01	82.5%	<0.01	
High	1	-0.60 (-0.86 to -0.34)	<0.01	0%	1.00	
**Race**						0.71
Asian	2	-0.47 (-0.65 to -0.29)	<0.01	0%	0.41	
Non-Asian	5	-0.51 (-0.67 to -0.36)	<0.01	77.1%	<0.01	

Abbreviations: CI, confidence interval; CKD, chronic kidney disease; DD, dialysis-dependent; DM, diabetes mellitus; HF, heart failure; NDD, non–dialysis-dependent; RAASi, renin–angiotensin–aldosterone system inhibitors; RR, relative risk; sK^+, serum potassium; SZC, sodium zirconium cyclosilicate; WMD, weighted mean difference

Supplemental Table 4. Definitions of responders

No.	Study	Definition
1	Packham DK. [1]	Serum potassium levels maintained between 3.5 and 4.9 mEq/L.
2	Fishbane S. [2]	Predialysis serum potassium maintained within 4.0–5.0 mmol/L during at least 3 out of 4 hemodialysis sessions following the long interdialytic interval, without the need for rescue therapy.
3	Peacock WF. [3]	Serum potassium <6.0 mmol/L within 1–4 hours and <5.0 mmol/L at 4 hours post-treatment, with no rescue therapy administered.
4	Ni Z. [4]	Pre-dialysis serum potassium maintained within 4.0–5.0 mmol/L during at least 3 out of 4 long interdialytic interval visits, without rescue therapy.
5	Ash SR. [5]	Serum potassium between 3.5 and 5.0 mmol/L at the end of treatment, without the use of rescue therapy.

Supplemental Table 5. Definitions of hyperkalemia utilized across included studies

No.	Study	sK^+ cut off (mEq/L)
1	Pitt B. [6]	>5.5
2	Kosiborod M. [7]	≥5.1
3	Ash SR. [8]	>5.0
4	Peckham D. [1]	>5.0
5	Weir MR. [9]	≥5.5
6	Agarwal R. [10]	≥5.5
7	Fishbane S. [2]	>5.0
8	Kashihara N. [11]	≥5.1
9	Peacock WF. [3]	>5.0
10	Butler J. [12]	>5.5
11	Jaques DA. [13]	≥5.1 or ≥5.5
12	Ni Z. [4]	>5.5
13	Ash SR. [5]	>5.0
14	Kang J. [14]	>5.3
15	Middleton JP. [15]	≥5.5
16	Charytan DM. [16]	>5.5
17	Elsayed MM. [17]	>5.0
18	Kashihara N. [18]	≥5.5
19	Kashihara N. [19]	≥5.1

Supplemental Table 6. Definitions of the optimal range of serum potassium utilized across included studies

No.	Study	Definition
1	Kosiborod M. [7]	Serum potassium 3.5–5.0 mEq/L
2	Kashihara N. [11]	Serum potassium 3.5–5.0 mEq/L
3	Ni Z. [4]	Predialysis serum potassium 3.5–5.5 mEq/L measured at long interdialytic interval visits during the evaluation period
4	Charytan DM. [16]	Predialysis serum potassium 4.0–5.5 mEq/L

Supplemental Table 7. Sub-group analyses examining the effect of patiromer on serum potassium reduction at the end of study.

Subgroup analyses	No. of studies	WMD (95% CI)	P-values	Assessment of heterogeneity		P for interaction
				I^2 index	P-value	
**Participants with CKD**						**0.04**
<50%	1	-0.21 (-0.26 to -0.16)	<0.01	0%	1.00	
≥50%	6	-0.40 (-0.57 to -0.23)	<0.01	85.2%	<0.01	
**Participants with DM**						0.30
<50%	3	-0.27 (-0.48 to -0.06)	0.01	96.6%	<0.01	
≥50%	4	-0.46 (-0.76 to -0.16)	<0.01	88.1%	<0.01	
**Participants with HF**						0.50
<50%	4	-0.46 (-0.76 to -0.16)	<0.01	88.1%	<0.01	
≥50%	2	-0.33 (-0.57 to -0.10)	0.01	98.1%	<0.01	
**% RAASi use**						**0.01**
<50%	1	-0.08 (-0.34 to 0.18)	0.54	0%	1.00	
≥50%	2	-0.52 (-0.75 to -0.29)	<0.01	50.3%	0.16	
**Dialysis status**						**0.04**
NDD-CKD	6	-0.40 (-0.56 to -0.24)	<0.01	93.7%	<0.01	
DD-CKD	1	-0.08 (-0.34 to 0.18)	0.54	0%	1.00	
**Risk of bias**						0.70
Low	4	-0.32 (-0.61 to -0.04)	0.03	88.6%	<0.01	
Some concern	2	-0.43 (-0.93 to 0.06)	0.09	85.9%	0.01	
High	1	-0.45 (-0.48 to -0.42)	<0.01	0%	1.00	
**Race**						0.26
Asian	3	-0.40 (-0.74 to -0.06)	0.02	91%	<0.01	
Non-Asian	3	-0.40 (-0.68 to -0.12)	0.01	80%	0.01	
Mixed	1	-0.21 (-0.26 to -0.16)	<0.01	0%	1.00	

Abbreviations: CI, confidence interval; CKD, chronic kidney disease; DD, dialysis-dependent; DM, diabetes mellitus; HF, heart failure; NDD, non–dialysis-dependent; RAASi, renin–angiotensin–aldosterone system inhibitors; RR, relative risk; sK^+, serum potassium; SZC, sodium zirconium cyclosilicate; WMD, weighted mean difference

Supplemental Table 8. Results of meta-regression between variables and serum potassium reduction from sodium zirconium cyclosilicate and patiromer.

Variable	Beta	SE	95% CI	z	P-value	I^2 (%)	R^2 (%)
**Sodium zirconium cyclosilicate**							
**Serum K^+ reduction at the end of initial phase**							
Age of participants	-0.020	0.01	-0.045 to 0.005	-1.51	0.132	80.82	21.51
Study sample size	<0.001	<0.001	-0.0004 to 0.0007	0.47	0.641	84.46	0
Baseline serum K+	-0.222	0.29	-0.794 to 0.351	-0.76	0.448	84.65	0
Baseline eGFR	0.015	0.008	-0.0005 to 0.030	1.90	0.058	79.57	43.01
**Serum K^+ reduction at the end of maintenance phase**							
Age of participants	-0.005	0.013	-0.031 to 0.021	-0.35	0.725	72.63	0
Study sample size	<0.001	<0.001	-0.0004 to 0.0008	0.67	0.502	69.41	0
Baseline serum K+	-0.106	0.127	-0.356 to 0.144	-0.83	0.407	69.73	0
Baseline eGFR	0.003	0.010	-0.018 to 0.023	0.25	0.803	85.78	0
**Patiromer**							
**Serum K^+ reduction at the end of maintenance phase**							
Age of participants	0.029	0.073	-0.113 to 0.172	0.40	0.687	91.66	0
Study sample size	<0.001	<0.001	-0.0004 to 0.0007	0.51	0.610	86.48	0
Baseline serum K+	-0.056	0.171	-0.392 to 0.280	-0.33	0.743	94.05	0
Baseline eGFR	0.006	0.010	-0.0143 to 0.026	0.59	0.557	94.08	0

Supplemental Figure 1. Funnel plot displaying the standard error by the mean difference in serum potassium reduction following the initial phase of SZC treatment across individual studies. (P = 0.77)

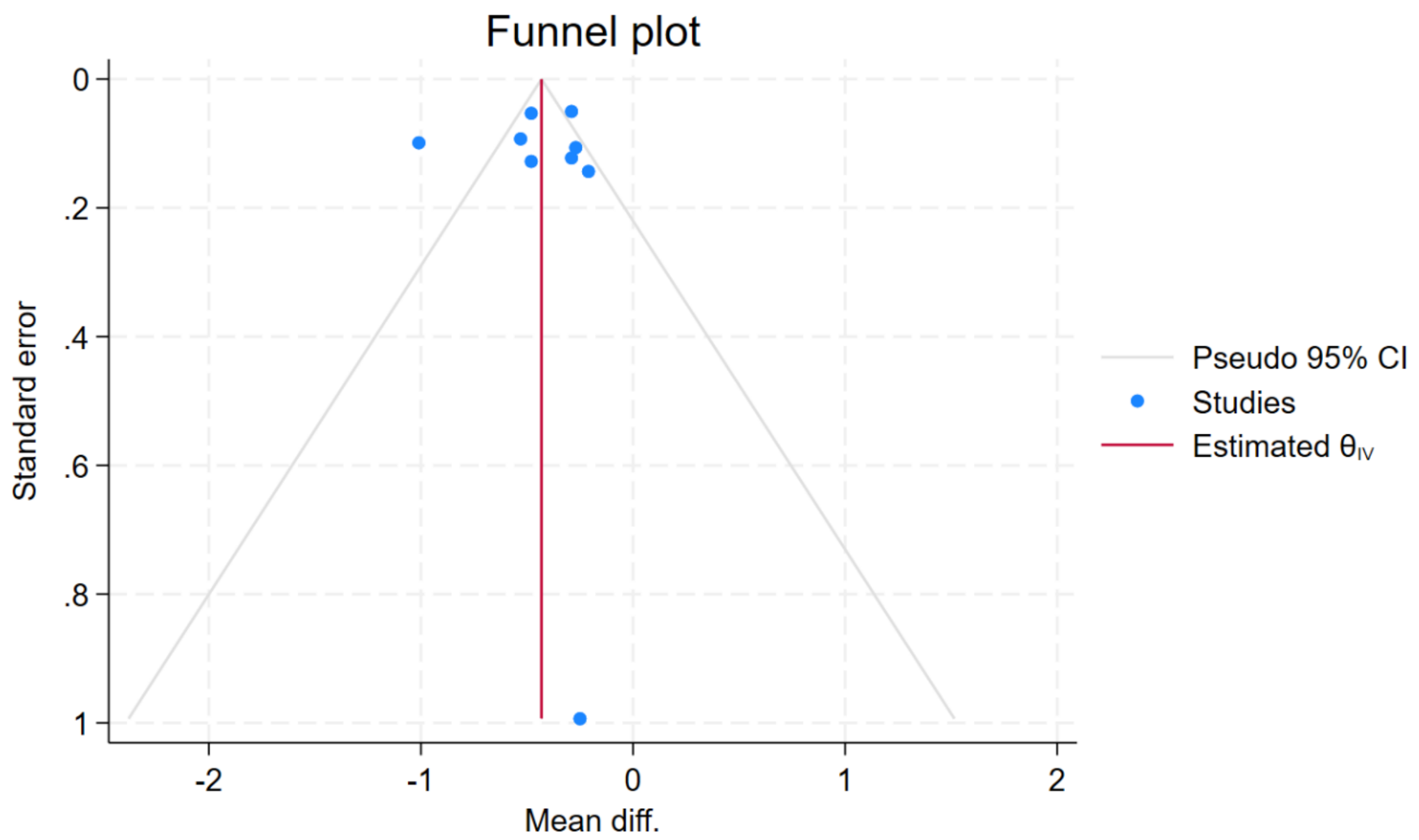

Supplemental Figure 2. Funnel plot displaying the standard error by the mean difference in serum potassium reduction following the maintenance phase of SZC treatment across individual studies (P=0.68).

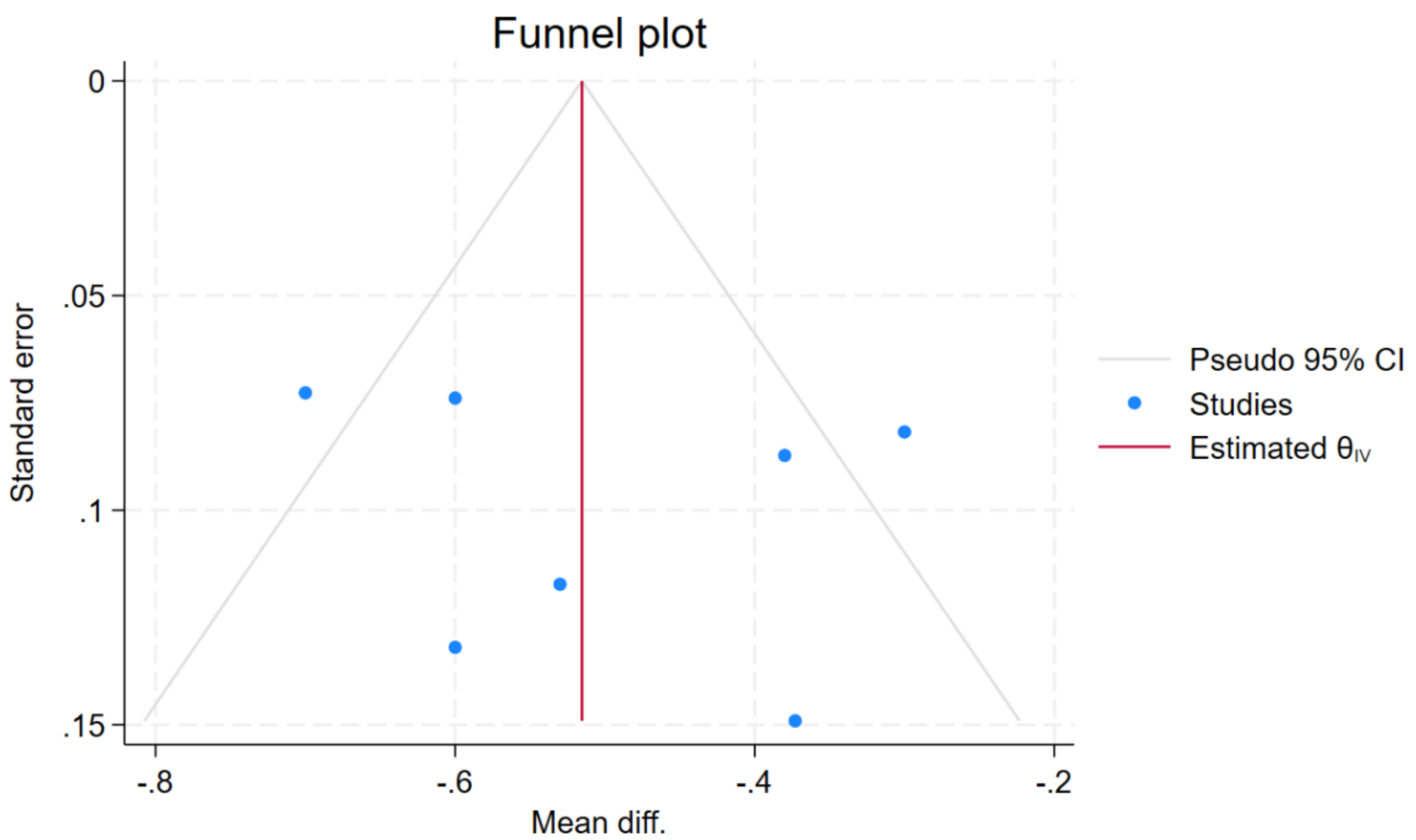

Supplemental Figure 3. Funnel plot displaying the standard error by the mean difference in serum potassium reduction following the maintenance phase of patiromer treatment across individual studies (P=0.50).

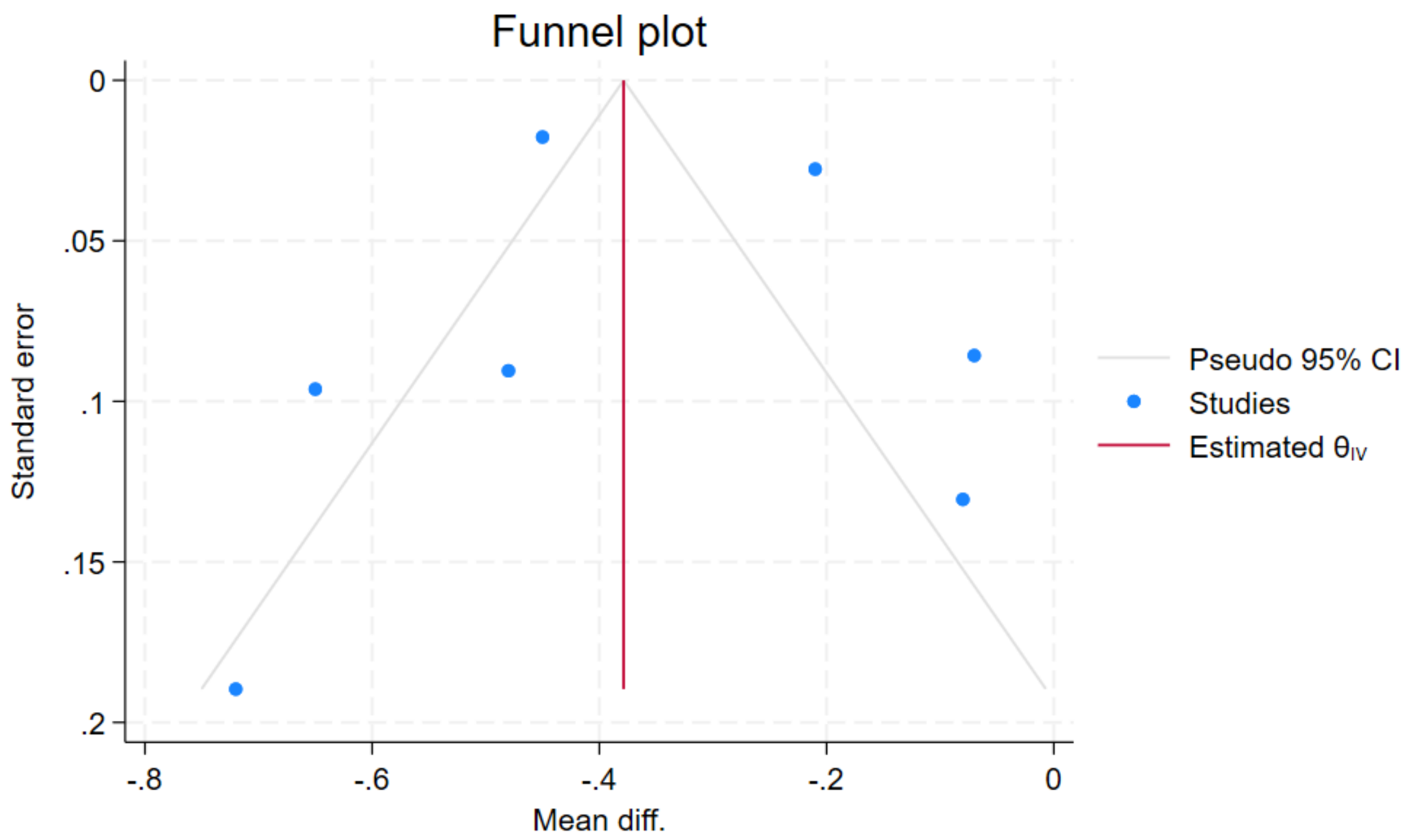

References

1. Packham DK, Rasmussen HS, Lavin PT, El-Shahawy MA, Roger SD, Block G, et al. Sodium zirconium cyclosilicate in hyperkalemia. N Engl J Med. 2015;372(3):222-31.

2. Fishbane S, Ford M, Fukagawa M, McCafferty K, Rastogi A, Spinowitz B, et al. A Phase 3b, Randomized, Double-Blind, Placebo-Controlled Study of Sodium Zirconium Cyclosilicate for Reducing the Incidence of Predialysis Hyperkalemia. J Am Soc Nephrol. 2019;30(9):1723-33.

3. Peacock WF, Rafique Z, Vishnevskiy K, Michelson E, Vishneva E, Zvereva T, et al. Emergency Potassium Normalization Treatment Including Sodium Zirconium Cyclosilicate: A Phase II, Randomized, Double-blind, Placebo-controlled Study (ENERGIZE). Acad Emerg Med. 2020;27(6):475-86.

4. Ni Z, Lu R, Xu X, Bian X, Zhou Z, Yang J, et al. DIALIZE China: A Phase IIIb, Randomized, Placebo-Controlled Study to Reduce Predialysis Hyperkalemia With Sodium Zirconium Cyclosilicate in Chinese Patients. Clin Ther. 2023;45(7):633-42.

5. Ash SR, Batlle D, Kendrick J, Oluwatosin Y, Kooienga L, Eudicone JM, et al. Sodium Zirconium Cyclosilicate in CKD, Hyperkalemia, and Metabolic Acidosis: NEUTRALIZE Randomized Study. Kidney360. 2024;5(6):812-20.

6. Pitt B, Anker SD, Bushinsky DA, Kitzman DW, Zannad F, Huang IZ. Evaluation of the efficacy and safety of RLY5016, a polymeric potassium binder, in a double-blind, placebo-controlled study in patients with chronic heart failure (the PEARL-HF) trial. Eur Heart J. 2011;32(7):820-8.

7. Kosiborod M, Rasmussen HS, Lavin P, Qunibi WY, Spinowitz B, Packham D, et al. Effect of sodium zirconium cyclosilicate on potassium lowering for 28 days among outpatients with hyperkalemia: the HARMONIZE randomized clinical trial. Jama. 2014;312(21):2223-33.

8. Ash SR, Singh B, Lavin PT, Stavros F, Rasmussen HS. A phase 2 study on the treatment of hyperkalemia in patients with chronic kidney disease suggests that the selective potassium trap, ZS-9, is safe and efficient. Kidney Int. 2015;88(2):404-11.

9. Weir MR, Bakris GL, Bushinsky DA, Mayo MR, Garza D, Stasiv Y, et al. Patiromer in patients with kidney disease and hyperkalemia receiving RAAS inhibitors. N Engl J Med. 2015;372(3):211-21.

10. Agarwal R, Rossignol P, Romero A, Garza D, Mayo MR, Warren S, et al. Patiromer versus placebo to enable spironolactone use in patients with resistant hypertension and

chronic kidney disease (AMBER): a phase 2, randomised, double-blind, placebo-controlled trial. Lancet. 2019;394(10208):1540-50.

11. Kashihara N, Nishio T, Osonoi T, Saka Y, Imasawa T, Ohtake T, et al. Correction of serum potassium with sodium zirconium cyclosilicate in Japanese patients with hyperkalemia: a randomized, dose-response, phase 2/3 study. Clin Exp Nephrol. 2020;24(12):1144-53.

12. Butler J, Anker SD, Lund LH, Coats AJS, Filippatos G, Siddiqi TJ, et al. Patiromer for the management of hyperkalemia in heart failure with reduced ejection fraction: the DIAMOND trial. Eur Heart J. 2022;43(41):4362-73.

13. Jaques DA, Stucker F, Ernandez T, Alves C, Martin PY, De Seigneux S, et al. Comparative efficacy of patiromer and sodium polystyrene sulfonate on potassium levels in chronic haemodialysis patients: a randomized crossover trial. Clin Kidney J. 2022;15(10):1908-14.

14. Kang J, Li S, Su J, Xiao Z, Zhang S, Liu S, et al. Effect of sodium zirconium cyclosilicate on hyperkalemia after parathyroidectomy in secondary hyperparathyroidism patients with maintenance hemodialysis: A randomized trial. Medicine (Baltimore). 2024;103(52):e40917.

15. Middleton JP, Sun S, Murray S, Davenport CA, Daubert JP. Randomized Trial of Patiromer on Efficacy to Reduce Episodic Hyperkalemia in Patients with ESKD Treated With Hemodialysis. Kidney Int Rep. 2024;9(11):3218-25.

16. Charytan DM, Winkelmayer WC, Granger CB, Middleton JP, Herzog CA, Chertow GM, et al. Effects of dialysate potassium concentration of 3.0 mmol/l with sodium zirconium cyclosilicate on dialysis-free days versus dialysate potassium concentration of 2.0 mmol/l alone on rates of cardiac arrhythmias in hemodialysis patients with hyperkalemia. Kidney Int. 2025;107(1):169-79.

17. Elsayed MM, Abdelrahman MA, Sorour AM, Rizk IG, Hassab MAA. Sodium zirconium cyclosilicate versus sodium polystyrene sulfonate for treatment of hyperkalemia in hemodialysis patients: a randomized clinical trial. BMC Nephrol. 2025;26(1):227.

18. Kashihara N, Okada H, Suzuki Y, Iwamoto T, Yasutomi M, Matsui M, et al. Efficacy and safety of patiromer for hyperkalemia: a randomized, placebo-controlled phase 3 study. Clin Exp Nephrol. 2025;29(7):899-911.

19. Kashihara N, Kumeda Y, Higashino Y, Maeda Y, Kaneko Y, Kanai H, et al. Efficacy and safety of patiromer for non-dialysis and dialysis patients with hyperkalemia: the randomized, placebo-controlled and long-term study. Clin Exp Nephrol. 2025;29(5):548-59.
